# A multi-center randomized, controlled, open-label trial evaluating the effects of eosinophil-guided corticosteroid-sparing therapy in hospitalised patients with COPD exacerbations – The CORTICO steroid reduction in COPD (CORTICO-COP) study protocol

**DOI:** 10.1186/s12890-017-0458-7

**Published:** 2017-08-15

**Authors:** Pradeesh Sivapalan, Mia Moberg, Josefin Eklöf, Julie Janner, Jørgen Vestbo, Rasmus Rude Laub, Andrea Browatzki, Karin Armbruster, Jon Torgny Wilcke, Niels Seersholm, Ulla Møller Weinreich, Ingrid Louise Titlestad, Helle Frost Andreassen, Charlotte Suppli Ulrik, Uffe Bødtger, Thyge Lynghøj Nielsen, Ejvind Frausing Hansen, Jens Ulrik Stæhr Jensen

**Affiliations:** 1 Department of Internal Medicine, Herlev and Gentofte University Hospital, Hellerup, Denmark; 2 Department of Respiratory Medicine, Bispebjerg University Hospital, Copenhagen, Denmark; 3 Division of Infection, Inflammation and Respiratory Medicine, University of Manchester, Manchester, UK; 4 Department of Pulmonary Medicine, Hvidovre University Hospital, Hvidovre, Denmark; 5 Department of Pulmonary and Infectious Diseases, North Zealand University Hospital, Hilleroed, Denmark; 6 Department of Respiratory Diseases, Aalborg University Hospital, Aalborg, Denmark; 7 Department of Respiratory Medicine, Odense University Hospital, University of Southern Denmark, Odense, Denmark; 8 Department of Respiratory Medicine, Naestved University Hospital, Naestved, Denmark; 9 CHIP, Department of Infectious Diseases, University of Copenhagen, Rigshospitalet, Finsencentret, Copenhagen, Denmark

**Keywords:** Systemic corticosteroids, Copd, Exacerbations, Eosinophil-guided corticosteroid-sparing therapy, Randomized controlled trial, Aecopd, Gcp, Biomarker, Length of hospital stay

## Abstract

**Background:**

The most commonly applied treatment for acute exacerbations of chronic obstructive pulmonary disease (AECOPD) is a 5-day course of high-dose systemic corticosteroids. However, this treatment has not been shown to reduce mortality and can potentially have serious side effects.

Recent research has shown that, presumably, only a subgroup of COPD patients identifieable by blood eosinophil count benefit from a rescue course of prednisolone. By applying a biomarker-guided strategy, the aim of this study is to determine whether it is possible to reduce the use of systemic corticosteroids in AECOPD without influencing the outcome.

**Methods:**

This is an ongoing prospective multicenter randomized controlled open label trial comprising 320 patients with AECOPD recruited from four hospitals in Denmark. The patients are randomized 1:1 to either standard care or eosinophil-guided corticosteroid-sparing therapy where prednisolone is not administered if the daily blood sampling reveals an eosinophil level below 0.3 × 10^9^ cells/L. The primary endpoint is length of hospital stay within 14 days after recruitment. The secondary endpoints are treatment failure, 30-day mortality rate, COPD related re-admission rate, change in FEV_1_, and a number of adverse effect measures obtained within 3 months after the index hospitalisation date related to corticosteroid usage.

**Discussion:**

This will be a very large RCT providing knowledge about the effectiveness of individualized biomarker-guided corticosteroid therapy in hospitalised patients with AECOPD.

**Trial registration:**

Clinicaltrials.gov, NCT02857842, 02-august-2016. Clinicaltrialregister.eu: Classification Code: 10,010,953, 02-marts-2016.

## Background

Acute exacerbation of chronic obstructive pulmonary disease (AECOPD) often leads to hospitalization and is associated with a high mortality rate. AECOPD episodes lead to accelerated decline in lung function and have negative impact on physical activity and quality of life. Furthermore, AECOPD implies high socioeconomic costs [1]. For decades, systemic corticosteroids have been a cornerstone in management of moderate to severe AECOPD. However, the optimal approach as regards the duration of treatment is unknown [2]. The REDUCE study concluded that a 5-day course of systemic corticosteroids is noninferior to 14-days of treatment [3]. Based on this, the recent GOLD strategy document recommends five to seven days of systemic corticosteroid treatment for patients with AECOPD [4].

### Previous research

A more recent Cohrane review (13 studies contributed data, *n* = 1620) regarding AECOPD patients has shown that systemic corticosteroids (compared to placebo) reduce the risk of treatment failure (number needed to treat (NNT) = 9; 95% confidence interval (CI) 7 to 14). Treatment failure was defined as patients who within 30 days required hospitalization or emergency room visits or patients requiring add-on pharmacological maintenance therapy. The risk of recurrence of AECOPD was also reduced within the first month (hazard ratio (HR) 0.78; 95% CI 0.63 to 0.97), whereas no difference in AECOPD recurrence was observed during the following 3 months [3].

Lung function measured up to 72 h after first administration of systemic corticosteroids showed significant improvement in forced expiratory volume in 1 s (FEV_1_) in the corticosteroid group (mean difference (MD) 140 mL; 95% CI 90 to 200), whereas this difference was not observed at later time-points. No difference was observed in mortality (odds ratio (OR) 1.0; 95% CI 0.60 to1.66). The total length of hospital stay (LOS) was shorter in the corticosteroid-treated group (MD −1.22 days; 95% CI -2.3 to −0.2), whereas there was no difference in the LOS in intensive care unit. The risk of steroid induced side effects was, as expected, increased (OR 2.33; 95% CI 1.59 to 3.43) in the corticosteroid-treated group compared with the control group; number needed to harm (NNH) = 6; 95% CI 4 to 10). Meanwhile, the proportion of side effects in the corticosteroid group (48.1%) was considerably higher than the control group (28.5%). The risk of hyperglycemia was increased (OR 2.79; 95% CI 1.86 to 4.19) and the absolute risk was 28.2% [3]. Others have identified serious psychiatric side effects (depression, mood changes, psychosis) and serious somatic side effects such as hypertension, ulcers, secondary adrenal insufficiency, diabetes, osteoporosis and increased risk of bone fractures in patients exposed to long-term systemic corticosteroids [5–7].

Earlier, the inflammatory process in patients with AECOPD was believed to be homogeneous, primarily neutrophilic. However, recent studies have shown that both inflammation [8, 9] and ethiology [10–12] are heterogeneous. It has been demonstrated that a subgroup of patients with AECOPD have eosinophilic inflammation [13]. Specific attention on biological clusters and biomarkers of these have resulted in an increased understanding of the differentiated inflammatory mechanisms that exist in AECOPD [14]. Examinations of sputum from the airways in smokers have increased this understanding further [15].

A small randomized study (*n* = 109) of COPD patients with moderate exacerbations has indicated that blood eosinophil-guided corticosteroid treatment might reduce the use of systemic corticosteroids in exacerbations by 49% without simultaniously worsening of symptoms and increasing risk of treatment failure compared with standard care [16]. Furthermore, patients with initial low eosinophil count more often experienced treatment failure if they had received corticosteroid therapy rather than if they had not (15% treatment failure in prednisolone group, 2% treatment failure in corticosteroid-saving group, *p* = 0.04). However, the study had no impact on current recommendations due to the limited sample size. Larger cohort studies documented that diagnosed COPD patients with daily symptoms have more than 3-fold increased risk for AECOPD if they have an eosinophil count ≥0.34 × 10^9^ cells/L [17].

So, the question that remains to be answered based on the currently available studies is whether the clinical effects of systemic corticosteroid treatment in hospitalised patients with AECOPD can be achieved via a more targeted and individualized eosinophil-guided treatment rather than the current “one-size fits all” treatment. To our knowledge, no randomized clinical trials have yet examined this field. This study will address this important question. The aim is to determine whether it is possible to reduce the use of systemic corticosteroids in AECOPD without influencing the outcome by applying a biomarker-guided strategy. Additionally, the study will explore whether this strategy reduces some of the most frequent side effects that occur with the current standard treatment.

## Methods

### Objective

The overall objective is to determine whether a prednisolone regimen with lower accumulated doses in hospitalised patients with AECOPD have noninferior clinical outcome than when applying standard care (SC). Based on a sufficiently sized (powered) clinical study, the aim is to determine if there is no relevant increase in LOS for AECOPD patients receiving eosinophil guided prednisolone-sparing therapy compared to SC. The primary endpoint of this analysis is LOS defined as the time from hospital admission to hospital discharge within 14 days after recruitment. The secondary objective is to determine if the clinical outcome for patients receiving eosinophil guided corticosteroid-sparing therapy will not be less favourable compared to SC. The following endpoints will be included when assessing the clinical outcome: 30-day mortality rate, treatment failure, COPD related re-admission rate, changes in FEV_1_, changes in health-related quality-of-life measured by COPD Assessment Test (CAT), changes in level of dyspnoea using the modified Medical Research Council (mMRC) Dyspnoea Scale, cumulative systemic corticosteroid dose, the period between index AECOPD and the next AECOPD and a number of adverse effect measures between index hospitalization and 3 month follow-up. The adverse effect measures include hyperglycemia, osteopenia (Bone Turnover Markers: C-terminal telopeptide of type 1 collagen (CTX) and procollagen type 1 N propeptide (P1NP)), osteoporotic fractures, dyspepsia or ulcer complications (gastrointestinal bleeding), onset of diabetes mellitus (defined as HbA1c ≥ 48 mmol/mol) or worsening of diabetes mellitus (defined as initiation/intensification of anti-diabetic treatment), increase in Body Mass Index and new infections treated with antibiotics since study entry.

### Study design, randomization and intervention

The study will be conducted as a prospective, multicenter, randomized, controlled, open-label study in hospitalised patients with AECOPD. In total, 320 patients are expected to be included in the project within 24 months. Patients will be randomly assigned in a 1:1 fashion to either an intervention group where guidance of prednisolone treatment is based on daily eosinophil count (“eosinophil-guided corticosteroid-sparing therapy”) or a standard group in which guidance of prednisolone is based on an existing guideline, see below. Pre-stratified block randomization with blocks of varying (and blinded) size will be applied to ensure equal distribution of patients on site (four different pulmonary departments in Denmark) and age (above or below 70 years of age). The study was monitored according to Good Clinical Practice (GCP) by the GCP unit at Bispebjerg University Hospital, Copenhagen, Denmark.

Patients will be randomized to one of the two treatment arms:

- Standard Care (SC) group: Intravenous methylprednisolone 80 mg on the first day followed by 37.5 mg of prednisolone tablets daily for 4 days.
- Intervention group: Intravenous methylprednisolone 80 mg, followed by prednisolone tablet 37.5 mg daily (maximum of 4 days in all) on days where the blood eosinophil count is ≥0.3 × 10^9^ cells/L. On days with eosinophil count <0.3 × 10^9^ cells/L systemic corticosteroid treatment will not be administered.

If a patient is discharged during the treatment period, a treatment based on the last measured eosinophil count will be prescribed for the remaining days within the 5 day-period.

#### Selection of participants

**Fig. 1:**
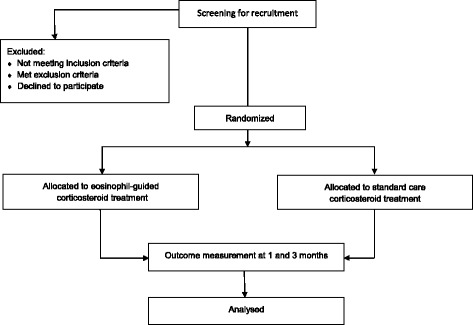
Flowchart of patients through the study

All consecutive patients admitted with AECOPD in participating centres will be considered for study enrolment (Fig. 1). Fulfilling the inclusion and exclusion criteria, patients will be invited to participate in the project. The aim is to enroll a sample representative of hospitalized patients with AECOPD in Denmark. Subjects on chronic systemic corticosteroids will be eligible for inclusion if they are receiving systemic corticosteroids corresponding to 5–10 mg daily. Patients receiving non-invasive ventilation (Continuous Positive Airway Pressure/Bilevel Positive airway pressure) were only recruited if they were admitted at one of the four recruiting departments.

### Inclusion criteria

- Hospitalized patients with AECOPD within 24 h after admission
- Age ≥ 40 years
- Spirometry-verified COPD (defined as postbronchodilator FEV_1_/FVC ≤ 0.70)

### Exclusion criteria

- Self-reported physician diagnosed asthma
- Life expectancy less than 30 days
- Severe COPD exacerbation requiring invasive ventilation or admission to ICU
- Allergy to systemic corticosteroids
- Severe mental illness which is not controlled by medication
- People who are detained under the act on the use of coercion in psychiatry
- Severe language difficulties or inability to provide written informed consent
- Pregnancy and lactation
- Systemic fungal infections

### Measurements

All outcome measures will be recorded at baseline and at the one and three month follow up visits (Table 1). On the baseline visit (during hospitalization), the following variables will be noted: sociodemographic data (e.g. sex, age, height, weight, ethnicity), smoking history, pack-years, co-morbidities (including the presence of diabetes, heart failure, ischemic heart disease, atrial fibrillation, essential hypertension, hypercholesterolemia), employment status, support with activities of daily living at home, clinical symptoms (increased dyspnea, increased sputum volume, and increased sputum purulence (Anthonisen criteria), increased cough), disease symptoms duration, body mass index (kg/m^2^), number of exacerbations in the past, atopy status, mean cumulative systemic corticosteroid dose (mg) 4 weeks before study entry, use of oxygen therapy, use of noninvasive mechanical ventilation, vital signs, adverse events to systemic corticosteroids, medication adherence by day 5, arterial blood gases and Ca^2+^, blood samples, findings from Chest X-ray and spirometry will be collected and stored on Case Report Forms in pseudoanonymised format at the treatment site (Table 1). Furthermore, patients will be requested to complete the CAT and mMRC Dyspnoea Scale. Follow-up visits will be conducted one and three months after discharge. If the patient does not attend the visit, the patient will be contacted for a new follow-up or - if not able to meet for an appointment at the hospital - a home visit by a doctor assigned to the project. At the one month and the three month follow up visits the following data will be assessed: BMI, HbA1c (only at the one month follow-up), spirometry values, CAT score, mMRC, CTX, P1NP, PTH (only at the three month follow-up), D-vitamin status (only at the three month follow-up), cumulative systemic corticosteroid dose, the period between index AECOPD and the next AECOPD, COPD related re-admission rate, dyspepsia or ulcer complications (gastrointestinal bleeding) and new infections treated with antibiotics since study entry.

**Table 1:** Data collected at baseline and follow-up visits

Data collected	Study period		
	Baseline	1-month	3-month
Demographics	X		
Daily blood glucose measurements	X		
Daily leukocyte differential count^a^	X		
Arterial blood gases and Chest X-ray	X		
Testing for diabetes (HbA1c)	X	X	
Spirometry	X (day 1 + day 3)	X	X
Height measurement & vital signs	X		
Weight measurement	X	X	X
Vitamin D and PTH levels	X		X
COPD Assessment Test (CAT)	X	X	X
Medical Research Council Dyspnoea Scale	X	X	X
Bone Turnover Markers (CTX, P1NP)	X	X	X
Questionnaire on general health condition		X	X
Pregnancy test	X		

^a^Completed by patients in the intervention group only

Collection of data and storage will comply with Good Clinical Practice guidelines as defined by the International Conference on Harmonisation guidelines [18]. Analysis of anonymised data will be conducted by the principal investigator with assistance from site investigators and supervisors.

### Medication adherence

All patients discharged during the treatment period (< 5 days) will be contacted in order to confirm whether the prescribed medication has been taken. This is to control for adherence with prescribed medication and to keep an accurate record of doses of medication for each patient.

### Withdrawal of study

In general, no subject should be removed from the study for a protocol violation prior to confirmation by the coordinating investigator. A patient is only to be withdrawn from the study if the participant explicitly asks for withdrawal.

### Size of the study population and statistical considerations

Data will be analysed using intention-to-treat (ITT) principles, including all the data available regardless of whether the intervention is completed. The aim of the ITT analysis is also to provide unbiased comparisons among groups and avoid the effects of dropout and handle patients who deviates from the protocol, e.g. intubated patients during their hospitalization.

Patients in SC will be compared to patients with eosinophil guided prednisolone sparing therapy (Intervention group). The mean LOS (μ = 8) and standard deviation (σ = 3.81 days) after a hospitalization for AECOPD is estimated based on previous studies comparing systemic corticosteroids versus placebo [3, 19]. A two-sided 95%-confidence interval will be computed for the difference in LOS in the intervention group minus standard care group. We will accept a null hypothesis if the LOS in the intervention group does not exceed 1.2 days of the average LOS in the SC arm. The probability that the study will detect a treatment difference is 80% at a two-sided 5% significance level. This provides a sample size of 320 subjects. Sample-size calculation has been performed by using SAS software (version 9.4).

The results are expressed in mean (days) and SD if data is normally distributed, or as median (IQR). Data will be analysed using SAS software.

### Trial Steering Committee & Data Safety Monitoring Board

A Trial Steering Committee has been established. We will meet twice a year with the Committee to advice the research team. Chair: MD, PhD Jens Ulrik Stæhr Jensen

A Data Safety Monitoring Board are in place to closely and independently monitor the safety of the CORTICO-COP study.

### Publication of test results

The test results will be published regardless of whether they are positive, negative or in-conclusive. Publication in international peer-reviewed scientific journals is planned accompanied by parallel publications in Danish Medical Journal. We aim to publish in high impact scientific journals. Results that cannot be published in peer-reviewed journals will be published on www.coptrin.eu and congresses in the form of posters and oral presentations.

## Discussion

COPD is a heterogeneous disease and is, by that, also associated with different responses to systemic corticosteroids for acute worsening of the disease. Although systemic corticosteroids improve some clinical outcomes in AECOPD - such as LOS, FEV_1_ on day 3, treatment failure and re-exacerbation rate the first month - the magnitude of benefit is small and probably restricted to subgroups of patients [3].

The novel aspect of this study is to investigate non-inferiority of eosinophilic guided corticosteroid-sparing therapy against the current standardized treatment of 5 days to all AECOPD patients. The study investigates whether the accumulated dose of systemic corticosteroid treatment during admissions for AECOPD can be reduced, including the presumed side effects, while simultaneously attaining the optimal treatment effect. Studies have shown that systemic corticosteroids improve outcome in exacerbations associated with either serum eosinophil counts equal to or above 2% [16] or eosinophil counts equal to or above 0.34 10^9^ cells/L [20]. However, on the other hand, this therapy might be detrimental in those with lower eosinophil counts [16]. A recent retrospective study has found that blood eosinophil-positive (eosinophilia ≥2%) severe AECOPD require a lower daily dose of systemic corticosteroids during hospitalization compared to blood eosinophil-negative (eosinophilia <2%) AECOPD. Thereby, the study concludes that the group with blood eosinophil-positive severe AECOPD respond better to systemic corticosteroids. Moreover, the former group had a shorter LOS (geometric mean 8.9 ± 1.5 versus 11.3 ± 1.5 days, respectively; *p* = 0.028) [21]. The result was supported by another sub-group analysis within a large randomized clinical trial which found shorter LOS in the group with eosinophilic (blood eosinophil count ≥200 cells/μL and/or ≥2% of the total leukocyte count) associated exacerbations (mean (range) 5.0 (1–19) vs. 6.5 (1–33), *p* = 0.015) following treatment with systemic corticosteroids [22].

Blood eosinophil count appears to be a promising biomarker that can be applied in the clinic to predict treatment response in patients with AECOPD. This could be beneficial by improving clinical outcome, decrease side effects and reduce any overuse of systemic corticosteroids [21]. The study will contribute with data to make clinical decisions on how to rationally individualize prednisolone treatment in hospitalized patients with AECOPD with the aim of optimizing treatment effect and reducing side effects.

### Trial status

Patient recruitment commenced in August 2016 and is ongoing.

## Abbreviations

AECOPD: Acute exacerbation of Chronic obstructive pulmonary disease
CAT: COPD Assessment Test
COPD: Chronic obstructive pulmonary disease
CRF: Case Report Form
FEV_1_: Forced expiratory volumen in 1. Second
FEV1/FVC ratio: also called Tiffeneau-index
FVC: Forced vital capacity;
GCP: Good Clinical Practice
GOLD: Global initiative for Chronic Obstructive Lung Disease
mMRC: Modified Medical Research Council
SC: Standard care
